# Pro-inflammatory markers and fatigue in patients with depression: A case-control study

**DOI:** 10.1038/s41598-020-66532-6

**Published:** 2020-06-11

**Authors:** Bruno Pedraz-Petrozzi, Elena Neumann, Gebhard Sammer

**Affiliations:** 1 Cognitive Neurosciences at the Center for Psychiatry and Psychotherapy, Justus-Liebig University, Giessen, Hessen Germany; 2 Internal Medicine and Rheumatology, Campus Kerckhoff, Justus-Liebig University, Bad Nauheim, Hessen Germany; 3 Faculty of Psychology and Sports Science, Justus-Liebig University, Giessen, Hessen Germany

**Keywords:** Human behaviour, Cognitive neuroscience, Molecular neuroscience, Neuroimmunology, Cytokines, Neuroimmunology, Fatigue, Depression

## Abstract

The aim of this work was to investigate differences between depressed subjects (PG) and non-depressed healthy control participants (HCG) with regard to fatigue dimensions and inflammation. For this purpose, 43 participants in the PG and 51 participants in the HCG were included in the study. IL-6, IL-1β, TNF-α, IFN-γ, and CRP were assessed in venous blood samples. Fatigue and depression were assessed using the FIS-D and BDI-FS questionnaires. Main results showed higher BDI-FS values in PG. Moreover, PG showed mean differences for fatigue dimensions when compared to the HCG. For the pro-inflammatory markers, a moderate group effect was found between PG and HCG which was mainly caused by IL-6. Correlations between TNF-α and BDI-FS, TNF-α and cognitive fatigue, TNF-α and psychosocial fatigue were found within the PG. In the HCG, correlations were found between IL-6, TNF-α and somatic fatigue, as well as IL-6 and cognitive fatigue. Significant correlations were found between the psychological variables in both groups. All results were controlled for the confounding variables *gender*, *age*, *BMI* and multiple comparisons. These results suggest the presence of inflammation in both depression and fatigue. However, each correlates with different pro-inflammatory parameters, suggesting a biological heterogeneity.

## Introduction

Depression is a mental health disorder, which is present in 27% of the world population and it is associated with a high DALY-metric^1,2^. The clinical features are predominantly lowering of mood, reduction of energy, and decrease in activity^3^. Among other symptoms, marked tiredness after even minimum effort is common^3^.

Well regarded models for depression frequently involve a stress-induced change in behavior. According to these models, stress activates different hypothalamic regions, which promotes higher concentrations of pro-inflammatory cytokines^4,5^. Two animal studies in mice have shown that higher levels of pro-inflammatory cytokines can lead to memory deficits and depressive mood symptoms^6,7^. This statement was corroborated in two studies who demonstrated a relationship between IL-1β and depressive disorders^8^ as well as with cognitive performance^9^.

Another consequence of the stress caused by overactivation of the hypothalamus is fatigue^5^, which it has been described as a diminished ability to initiate or maintain activity or to maintain attention^10,11^. Fatigue is considered either a common nonspecific symptom or a syndrome and until today there is no uniform definition. To reach a possible definition, two reviews from *Brurberg et al*. and *Watanabe et al*. proposed an dimensional approach for fatigue^12,13^, which include three important dimensions: somatic (e.g. musculoskeletal pain/tiredness), psychosocial (e.g. restrained social activity due to fatigue or mood impairment), and cognitive complains (e.g. decreased ability to maintain attention, memory impairment)^12,13^.

Following this dimensional approach, many conditions were associated with different dimensions of fatigue, including: drugs, deficiency diseases (i.e. iron-deficiency anemia), depression, anxiety disorders, burnout and chronic fatigue syndrome^14,15^. Interestingly, other conditions that also present fatigue are related strongly to inflammation as well (e.g. tumor-related fatigue, organ failure, chronic infectious diseases, endocrine diseases and autoimmune diseases, etc.)^14,15^. For instance, patients with chronic diseases such as rheumatoid arthritis^16^ or chronic kidney disease^17^ develop a persistent somatic and psychosocial fatigue that impairs the ability to perform daily activities. Additionally, it was demonstrated in patients with chronic kidney disease that higher levels of pro-inflammatory cytokines (i.e., IL-6) were associated with fatigue measured with the SF-36, which included mostly elements of the psychosocial and somatic fatigue dimensions^17^.

The presence of inflammation on perceived fatigue^18^ indicates possible dysregulation of the hypothalamic axis^19^. Such a deregulation also occurs in depressive disorders^20,21^, where fatigue is a common clinical manifestation. This has been shown in two studies in which higher cytokine levels and fatigue were correlated in patients with depressive symptoms^21,22^ and one study with healthy patients^23^. To our knowledge, however, there are no human studies on primary depression that relate inflammation to the above-mentioned fatigue dimensions.

To contribute to the evidence line for depression, which shows differences and possible correlations between fatigue and pro-inflammatory mechanisms in depression^19,24–26^, the main objectives of this study are (1) to examine whether the fatigue dimensions scores differ between participants with depression and non-depressed healthy control subjects, (2) to investigate whether the concentration of certain pro-inflammatory markers differ between the two groups, (3) to test whether there is a positive correlation between inflammation and the depression severity scores, and finally (4) the investigation of positive correlations between the values of fatigue dimensions and inflammation in participants with depression compared to non-depressed healthy control subjects.

## Results

### General characteristics of the sample

Baseline laboratory and clinical characteristics of the 94 participants included in the study are listed in Table 1. The sample consisted of about twice as many women as men. However, the ratio of men and women did not differ per group (Table 1). The age differed between the groups by about 1 year (median) with the IQR being slightly higher in the depression group (Table 1). More smokers were found in the patient group (*p* = 0.06, Cramer’s V = 0.21).

**Table 1:** Sample size description - General data.

Characteristic	All subjects (n = 94)	PG (n = 43)	HCG (n = 51)	P	ES
Age (in years)	24.50 (30.25–22.00; 8.25)	25 (36.00–22.00; 14.00)	24 (28.00–22.00; 6.00)	0.19	0.02
Gender (male: female)	31:63	12:31	19:32	0.38	0.10
BMI (kg/m^2^)	23.04 (26.32–21.21; 5.11)	23.72 (27.64–21.26; 6.38)	22.69 (25.46–21.09; 4.37)	0.57	0.003
Smoking status (yes: no)	16:78	11:32	05:46	0.06	0.21
Primary cause of DE					
*Mild DE*		1			
*Moderate DE*		9			
*Severe DE*		7			
*Dysthymia*		1			
*Recurrent DE*		2			
*DE NOS*		15			
*DE secondary to BD*		2			
*DE secondary to PTSD*		2			
*DE secondary to AD*		1			
*DE secondary to BPD*		1			
*DE secondary to APD*		1			
*Mixed DE with Anxiety*		1			

For variables age and BMI: U-Mann-Whitney-Test for group differences was computed. Effect sizes (Eta-squared) are reported. One-tailed-p-values (p < 0.05) are flagged (*).

For variables gender and smoking status: Fisher exact test for group differences was computed. Effect sizes (Cramer’s V) are reported. One-tailed-p-value (p < 0.05) are flagged (*).

Abbreviations: PG = patient group; HCG = healthy control group; P = p-value; ES = Effect size; BMI = Body-Mass-Index; DE = Depressive Episode; NOS = Not otherwise specified; BD = Bipolar disorder; PTSD = Post-traumatic stress disorder; AD = Adjustment disorder; BPD = Borderline personality disorder; APD = Avoidant personality disorder.

For variables age and BMI: U-Mann-Whitney-Test for group differences was computed. Effect sizes (Eta-squared) are reported. One-tailed-p-values (p < 0.05) are flagged (*).

### Pro-inflammatory markers: Fatigue dimensions and Depression

Fatigue dimensions (COG-F, SOM-F, PSY-F) and BDI-FS scores were analyzed using a MANCOVA. The grouping factor was *status* (PG and HCG), covariates were the pro-inflammatory markers (Table 3). There was a significant difference between the PG and HCG groups in the combined dependent psychological variables after control of the pro-inflammatory markers (F_4,74_ = 30.38, Wilks’ Λ = 0.38, p < 0.001, η^2^_p_ = 0.62). Of all pro-inflammatory markers used as covariates, only IL-6 predicts fatigue and depression (F_4,74_ = 4.68, Wilks’ Λ = 0.80, p = 0.002, η^2^_p_ = 0.20) (Table 3). The univariate tests showed differences for all fatigue dimensions and BDI-FS values between the groups PG and HCG (COG-F: F_1,77_ = 62.59, p < 0.001; SOM-F: F_1,77_ = 54.83, p < 0.001; PSY-F: F_1,77_ = 114.17, p < 0.001; BDI-FS _TOT_: F_1,77_ = 69.09, p < 0.001). Univariate tests for the covariate IL-6 concentrations showed a significant correlation with each of the fatigue dimensions (COG-F: F_1,77_ = 14.97, p < 0.001; SOM-F: F_1,77_ = 14.29, p < 0.001; PSY-F: F_1,77_ = 14.49, p < 0.001), but not with BDI-FS_TOT_ (F_1,77_ = 1.46, p = 0.23).

**Table 2:** Clinical data from the sample size.

	Status	BDI-FS	COG-F	SOM-F	PSY-F	IL-6	TNF-α	IL-1β	IFN-γ	CRP
N	**HCG**	51	51	51	51	51	51	51	51	46
	**PG**	43	43	43	43	42	43	42	43	39
Mean	**HCG**	2.04	11.47	7.53	15.26	2.43	13.03	2.60	12.96	1338.49
	**PG**	8.30	23.58	17.91	42.61	3.04	13.23	4.23	13.97	1127.74
25^th^ Percentile	**HCG**	0	7	3	6	1.38	8.95	0.84	9.97	229.90
	**PG**	5	18	13	29	1.36	8.23	0.75	10.36	184.00
Median	**HCG**	1	12	6	15	2.17	11.83	1.38	12.12	610.66
	**PG**	8	23	18	47	2.85	10.38	0.90	12.65	458.74
75^th^ Percentile	**HCG**	3	15	12	23	3.45	17.03	2.63	15.44	2429.00
	**PG**	11	30	22	52	4.10	16.38	4.37	15.44	1279.00
Standard deviation	**HCG**	2.48	6.36	5.98	9.54	1.34	4.77	3.69	3.98	1420.04
	**PG**	4.17	7.81	7.53	14.84	1.72	7.76	7.63	8.91	1548.52
Skewness	**HCG**	1.56	0.13	0.57	0.15	0.53	0.86	4.27	0.43	1.08
	**PG**	0.07	−0.30	−0.17	−0.41	0.57	3.14	3.14	4.82	1.98

Abbreviations: HCG = healthy controls group, PG = patient group, COG-F = Cognitive fatigue component scores (FIS-D); SOM-F = Somatic fatigue component scores (FIS-D); PSY-F = Psychosocial fatigue component scores (FIS-D); BDI – FS = Beck Depression Inventory – Fast Screening; IL-6 = Interleukin 6; TNF-α = Tumor necrosis factor alpha; IL-1β = Interleukin 1 Beta; IFN-γ = Interferon gamma; CRP = C-reactive Protein.

Abbreviations: HCG = healthy controls group, PG = patient group, COG-F = Cognitive fatigue component scores (FIS-D); SOM-F = Somatic fatigue component scores (FIS-D); PSY-F = Psychosocial fatigue component scores (FIS-D); BDI – FS = Beck Depression Inventory – Fast Screening; IL-6 = Interleukin 6; TNF-α = Tumor necrosis factor alpha; IL-1β = Interleukin 1 Beta; IFN-γ = Interferon gamma; CRP = C-reactive Protein.

**Table 3:** MANCOVA Analysis: Pro-inflammatory markers vs. perceived Fatigue and Depression (grouping factor: Status).

Multivariate Tests								
		Λ	F		*df*_*1*_	*df*_*2*_	*p*	η^2^_p_
Status		0.378	30.38		4	74	*< 0.001	0.62
IL-6		0.798	4.68		4	74	*0.002	0.20
TNF-α		0.915	1.72		4	74	0.15	0.09
IL-1β		0.938	1.22		4	74	0.31	0.06
IFN-γ		0.916	1.69		4	74	0.16	0.08
CRP		0.933	1.33		4	74	0.27	0.07
**Univariate Tests**								
	**DV**	**SS**		**df**	**MS**	**F**	***p***	**η**^**2**^_**p**_
Status	COG-F	2948.20		1	2948.20	62.59	*< 0.001	0.45
	SOM-F	2177.28		1	2177.28	54.83	*< 0.001	0.42
	PSY-F	16016.05		1	16016.05	114.17	*< 0.001	0.60
	BDI-FS_TOT_	821.98		1	821.98	69.09	*< 0.001	0.47
IL-6	COG-F	705.26		1	705.26	14.97	*< 0.001	0.16
	SOM-F	567.37		1	567.37	14.29	*< 0.001	0.16
	PSY-F	2032.54		1	2032.54	14.49	*< 0.001	0.16
	BDI-FS_TOT_	17.41		1	17.41	1.46	0.23	0.02
TNF-α	COG-F	7.89		1	7.89	0.17	0.68	0.002
	SOM-F	181.39		1	181.39	4.57	*0.04	0.06
	PSY-F	232.79		1	232.79	1.66	0.20	0.02
	BDI-FS_TOT_	0.48		1	0.48	0.04	0.84	0.001
IL-1β	COG-F	13.80		1	13.80	0.29	0.59	0.004
	SOM-F	37.88		1	37.88	0.95	0.33	0.01
	PSY-F	5.36		1	5.36	0.04	0.85	< 0.001
	BDI-FS_TOT_	9.48		1	9.48	0.80	0.38	0.01
IFN-γ	COG-F	3.44		1	3.44	0.07	0.79	0.001
	SOM-F	96.48		1	96.48	2.43	0.12	0.03
	PSY-F	21.97		1	21.97	0.16	0.69	0.002
	BDI-FS_TOT_	1.48		1	1.48	0.12	0.73	0.002
CRP	COG-F	47.83		1	47.83	1.02	0.32	0.01
	SOM-F	18.11		1	18.11	0.46	0.50	0.01
	PSY-F	40.76		1	40.76	0.29	0.59	0.004
	BDI-FS_TOT_	3.35		1	3.35	0.28	0.60	0.004
Residuals	COG-F	3627.15		77	47.11			
	SOM-F	3057.64		77	39.71			
	PSY-F	10802.10		77	140.29			
	BDI-FS_TOT_	916.14		77	11.90			

Two-tailed-p-values (p < 0.05) were flagged (*).

Abbreviations: Λ = Wilks’ Lambda; F = F-test value; p = p-value; DV = dependent variable; SS = Sum of Squares; df = degrees of freedom; MS = Mean square; η^2^p = partial eta-squared (effect size); IL-6 = Interleukin 6; TNF-α = Tumor necrosis factor alpha; IL-1β = Interleukin 1 Beta; IFN-γ = Interferon gamma; CRP = C-reactive Protein; COG-F = Cognitive fatigue component scores (FIS-D); SOM-F = Somatic fatigue component scores (FIS-D); PSY-F = Psychosocial fatigue component scores (FIS-D); BDI-FSTOT = Total scores of the Beck Depression Inventory – Fast Screening.

### Relationship between inflammation markers and age, gender and BMI

The confounding of age, gender and BMI with the fatigue dimensions as well as the BDI-FS values and also the pro-inflammatory markers were assessed with a MANCOVA. Here, too, the status was used as a grouping factor. The results of the analysis are shown in Table 4.

**Table 4:** MANCOVA Analysis: Pro-inflammatory markers, perceived Fatigue and Depression vs. BMI, age and gender (grouping factor: Status).

Multivariate Tests							
		Λ	F	*df1*	*df2*	*p*	η^2^_p_
Status		0.377	13.02	9	71	*< 0.001	0.62
Sex		0.728	2.94	9	71	*0.01	0.27
Age		0.718	3.09	9	71	*0.003	0.28
BMI		0.608	5.08	9	71	*< 0.001	0.39
**Univariate Tests**							
	**DV**	**SS**	***df***	**MS**	**F**	***p***	**η**^**2**^_**p**_
Status	IL-6	8.87	1	8.87	4.95	*0.03	0.06
	TNF-α	< 0.001	1	< 0.001	< 0.001	1.00	< 0.001
	IL-1β	21.55	1	21.55	0.71	0.40	0.01
	IFN-γ	44.39	1	44.39	0.90	0.35	0.01
	CRP	688712.56	1	688712.56	0.43	0.52	0.01
	COG-F	2948.20	1	2948.20	59.53	*< 0.001	0.43
	SOM-F	2177.28	1	2177.28	49.06	*< 0.001	0.38
	PSY-F	16016.05	1	16016.05	110.05	*< 0.001	0.58
	BDI-FS_TOT_	821.98	1	821.98	74.65	*< 0.001	0.49
Sex	IL-6	2.66	1	2.66	1.49	0.23	0.02
	TNF-α	70.99	1	70.99	1.72	0.19	0.02
	IL-1β	0.60	1	0.60	0.02	0.89	< 0.001
	IFN-γ	2.04	1	2.04	0.04	0.84	0.001
	CRP	8.68E+06	1	8.68E+06	5.40	*0.02	0.06
	COG-F	354.30	1	354.30	7.15	*0.01	0.08
	SOM-F	37.38	1	37.38	0.84	0.36	0.01
	PSY-F	168.35	1	168.35	1.16	0.29	0.01
	BDI-FS_TOT_	43.00	1	43.00	3.90	0.05	0.05
Age	IL-6	12.85	1	12.85	7.17	*0.01	0.08
	TNF-α	19.25	1	19.25	0.47	0.50	0.01
	IL-1β	28.68	1	28.68	0.94	0.34	0.01
	IFN-γ	26.13	1	26.13	0.53	0.47	0.01
	CRP	2.04E+06	1	2.04E+06	1.27	0.26	0.02
	COG-F	88.96	1	88.96	1.80	0.18	0.02
	SOM-F	179.91	1	179.91	4.05	0.05	0.05
	PSY-F	413.32	1	413.32	2.84	0.10	0.03
	BDI-FS_TOT_	26.67	1	26.67	2.42	0.12	0.03
BMI	IL-6	15.27	1	15.27	8.52	*0.01	0.10
	TNF-α	1.25	1	1.25	0.03	0.86	< 0.001
	IL-1β	42.31	1	42.31	1.39	0.24	0.02
	IFN-γ	26.17	1	26.17	0.53	0.47	0.01
	CRP	4.29E+07	1	4.29E+07	26.67	*< 0.001	0.25
	COG-F	49.36	1	49.36	1.00	0.32	0.01
	SOM-F	235.53	1	235.53	5.31	*0.02	0.06
	PSY-F	1056.92	1	1056.92	7.26	*0.01	0.08
	BDI-FS_TOT_	8.76	1	8.76	0.80	0.38	0.01
Residuals	IL-6	141.52	79	1.79			
	TNF-α	3256.53	79	41.22			
	IL-1β	2412.83	79	30.54			
	IFN-γ	3904.11	79	49.42			
	CRP	1.27E+08	79	1.61E+06			
	COG-F	3912.74	79	49.53			
	SOM-F	3506.04	79	44.38			
	PSY-F	11496.93	79	145.53			
	BDI-FS_TOT_	869.92	79	11.01			

Two-tailed-p-values (p < 0.05) were flagged (*).

Abbreviations: Λ = Wilks’ Lambda; F = F-test value; p = p-value; DV = dependent variable; SS = Sum of Squares; df = degrees of freedom; MS = Mean square; η^2^_p_ = partial eta-squared (effect size); BMI = Body-Mass-Index; IL-6 = Interleukin 6; TNF-α = Tumor necrosis factor alpha; IL-1β = Interleukin 1 Beta; IFN-γ = Interferon gamma; CRP = C-reactive Protein; COG-F = Cognitive fatigue component scores (FIS-D); SOM-F = Somatic fatigue component scores (FIS-D); PSY-F = Psychosocial fatigue component scores (FIS-D); BDI-FS_TOT_ = Total scores of the Beck Depression Inventory – Fast Screening.

There was a significant difference between the PG and HCG groups in the combined dependent variables after controlling the influence of age, gender and BMI (F_9,71_ = 13.02, Wilks’ Λ = 0.38, *p* < 0.001, η^2^_p_ = 0.62). All of the confounding variables showed a correlation with the dependent variables (*gender*: F_9,71_ = 2.94, *p* = 0.01, η^2^_p_ = 0.27; *age*: F_9,71_ = 3.09, *p* = 0.003, η^2^_p_ = 0.28, and *BMI*: F_9,71_ = 5.08, *p* < 0.001, η^2^_p_ = 0.39).

In a further analysis with univariate tests, the status showed significant differences between PG and HCG for IL-6 (F_1,79_ = 4.95, *p* = 0.03), for all the fatigue dimensions (COG-F: F_1,79_ = 59.53, *p* < 0.001; SOM-F: F_1,79_ = 49.06, *p* < 0.001; PSY-F: F_1,79_ = 110.05, *p* < 0.001) and for BDI-FS total scores (F_1,79_ = 74.65, *p* < 0.001). The other cytokines showed no significant status differences in univariate tests (Table 4).

Regarding the univariate tests involving the confounds, *gender* correlated with the acute-phase protein CRP (F_1,79_ = 5.40, *p* = 0.02) and with the cognitive fatigue (COG-F: F_1,79_ = 7.15, *p* = 0.01). *Age* correlated only with IL-6 concentrations (F_1,79_ = 7.17, *p* = 0.01). Finally, *BMI* correlated with IL-6 (F_1,79_ = 8.52, *p* = 0.01), CRP (F_1,79_ = 26.67, *p* < 0.001), somatic fatigue (SOM-F: F_1,79_ = 5.31, *p* = 0.02) and psychological fatigue (PSY-F: F_1,79_ = 7.26, *p* = 0.01).

### Correlations within groups between pro-inflammatory markers, fatigue scores and BDI-FS total scores

Pairwise correlations between all psychological variables and the five assessed pro-inflammatory markers’ concentrations were calculated separately for each group.

Based on the effects of age, gender and BMI on the proinflammatory markers, fatigue dimensions and overall BDI-FS values, this proportion of the variance was removed before the correlation analysis. The *p*-values of the correlation analysis were Bonferroni adjusted by setting the *p*-value threshold correction for 5 (i.e. the number of pro-inflammatory markers in this study) multiple comparisons (*p*_(α=0.05)_ = 0.01).

Different correlation patterns were found between the two groups. In the PG, TNF-α concentrations correlated with the COG-F scores (Spearman’s ρ = 0.36, *p* = 0.0095, CI95 [0.11, 1]) and with the PSY-F scores (Spearman’s ρ = 0.40, *p* = 0.004, CI95 [0.16, 1]). In the HCG, the cytokine IL-6 correlated with the COG-F scores (Spearman’s ρ = 0.34, p = 0.007, CI95 [0.12, 1]) and SOM-F scores (Spearman’s ρ = 0.37, p = 0.004, CI95 [0.15, 1]), and TNF-α correlated with the SOM-F scores (Spearman’s ρ = 0.34, p = 0.007, CI95 [0.12, 1]).

#### Correlations between the cytokine concentrations and the BDI-FS total scores

The BDI-FS scores correlated with TNF-α within the PG group (Spearman’s ρ = 0.47, p = 0.0008, CI95 [0.24, 1]), but not within HCG (Spearman’s ρ = −0.08, p = 0.70, CI95 [−0.30, 1]). All other pairwise correlations between cytokine correlations and BDI-FS have not exceeded the critical p-threshold.

#### Differences between depression and fatigue in the correlation analysis

We consider the question of whether fatigue can be distinguished from depression by examining the correlation patterns with the pro-inflammatory markers. A two-block stepwise regression model was performed. The first block considered the total BDI-FS values, the second block the fatigue values. This regression model was only calculated for the PG group, as this group presented a pro-inflammatory marker (i.e. TNF-α) that correlated with both depression and two fatigue dimensions (COG-F and PSY-F). The first model, which consisted only of block 1 (TNF-α x BDI-FS total scores), explained 10.9% of the TNF-α variance (R^2^ = 0.109, *p* = 0.037). When the second block with the COG-F values was added, no significant changes compared to the previous model were observed (R^2^ = 0.136, ΔR^2^ = 0.027, *p* = 0.267). Comparable results were obtained with PSY-F instead of COF-F (PSY-F: R^2^ = 0.133, ΔR^2^ = 0.024, *p* = 0.297).

## Discussion

### Differences between groups of depressed and non-depressed participants

The recruitment strategy was successful in terms of significantly higher BDI-FS values in the group of depressed study participants. All fatigue values, psychological, cognitive and somatic fatigue were also higher in depressed people. These results are consistent with the assumption that fatigue is a predominant symptom of depressive disorders^27^. A study evaluating excessive daytime sleepiness and fatigue in depressed patients showed significant differences in fatigue dimensions between patients and healthy controls^28^.

The results of fatigue dimensions and depression scores compared to the proinflammatory markers, which are shown in Table 3, show that the IL-6 concentrations differed between the fatigue dimensions, but not in the total depression scores. The finding that the IL-6 concentrations are different for all fatigue dimensions is consistent with the literature on the inflammatory components upon fatigue^17,19,29,30^.

Table 4 shows that the IL-6 concentrations differed between depressed and non-depressed participants. This result is consistent with two meta-analyses from human studies that reported significant differences of peripheral IL-6 levels in patients with depressive disorders compared to healthy controls^31,32^. However, the results obtained in Table 3 showed that all fatigue values, but not the total depression values, explained the IL-6 concentration differences. Therefore, fatigue could contribute to explain the pro-inflammatory components in depression. One plausible explanation for this could be that stress triggers HPA activation, which produces larger amounts of ACTH, which in turn stimulates increased cortisol production in the adrenal cortex and therefore promotes the release of IL-6 in the blood^4,33,34^. The released peripheral IL-6 could act directly on the adrenal cortex cells. Stimulation with IL-6 leads to an increased production of glucocorticoids in the blood and an increase in ACTH receptors in the adrenal cortex^35–37^. As a result of these IL-6-promoted effects in the CNS, the concentrations of glucocorticoids in depression could be increased^38–43^ and contributing finally to the development of several clinical manifestations of mood disorders, such as fatigue^43^.

Regarding the model processed in Table 4, confounding factors’ analysis showed interestingly an influence of *BMI* on somatic and psychosocial fatigue. This is consistent with studies showing that people with a higher BMI (e.g. obese adults) report a higher level of mental fatigue as well as insufficient physical activity and sleepiness due to fatigue^44^. Gender only had an impact on cognitive fatigue. This can possibly be seen in the context of the results of a German study on general population in which women showed higher scores for mental fatigue^45^.

The model presented in Table 4 also showed that the selected covariates (*gender*, *age* and *BMI*) had also remarkable influence. In terms of *gender*, CRP acute phase protein was different between men and women. Other studies have also found that CRP is slightly higher with age and gender^46^. As expected from the literature^47^, IL-6 increased with *age* and *BMI*. The latter also showed a correlation with CRP. This is also in line with the literature, which indicates mild inflammation due to a large amount of metabolic stress such as obesity^48–50^.

### Correlations within groups of depressed or non-depressed participants

Within each group, correlations between depression and all fatigue dimensions were on a similar and significant level. Only the correlation between depression and psychosocial fatigue in the patient group was definitely higher. These results support the commonly reported relationship between depression and fatigue on the subjective level. However, it should be borne in mind that the correlations are not strong enough to provide reliable evidence that depression and fatigue are the same (see, for example, the step-by-step regression with two blocks in Chapter 3.4). This is also supported by the correlation pattern of the pro-inflammatory markers with the psychological variables. Positive correlations of the pro-inflammatory cytokine TNF-α with psychosocial and cognitive fatigue as well as with the severity of the depression were found in depressed, but not in non-depressed participants. Interestingly, TNF-α was positively associated with somatic fatigue and IL-6 with somatic and cognitive fatigue in the non-depressed healthy controls group. A positive relationship between TNF-α and depressive symptoms^51^ has been reported earlier in the literature. In a study with systemic lupus erythematosus, a pro-inflammatory autoimmune disease, a higher TNF-α value was also associated with stronger depressive symptoms. This relationship is also supported by results that show increased gene expression in the TNF family in depressed subjects and negative correlations with cognitive efficiency^52^. When the results are viewed from the perspective of the effect of TNF-α on the CNS, TNF-α (like IL-6) is an inflammatory cytokine that is produced by macrophages^36,37^, that can actively cross the blood-brain barrier^53^, having a direct influence on the HPA^16^. This increases cortisol production and thus contributes to depression psychopathology^43^. In addition, TNF-α has a dose-dependent relationship to basal cortisol production. The more TNF-α is produced, the more cortisol is produced^54,55^. This dose-dependent relationship may also affect the relationship between TNF-α and the severity of the depression and, in this study, the BDI values.

Some studies have reported the relationship between cytokine concentrations and fatigue^19^. TNF-α is generally increased in chronic fatigue syndrome or fatigue^56^, in chronic kidney disease IL-6 is positively correlated with fatigue^17^.

TNF-α and IL-6 are both inflammatory cytokines that are produced by macrophages and increase cortisol levels in humans^57^. Glucocorticoids are not only involved in the development of muscle weakness, cognitive dysfunction, sleep disorders and a strong feeling of illness, but also in a changed mood. These clinical symptoms are more or less synonymous with fatigue and its dimensions, namely the somatic (e.g. muscle weakness), the cognitive (e.g. reduced attention and memory) and the psychosocial (e.g. high level of illness, sleepiness, fatigue) dimension.

The results of this study show different correlation patterns in the groups of depressed and non-depressed participants. The psychosocial fatigue component correlated with inflammation in the group of depressed participants, probably because this component is more common in depression^58–61^. On the other hand, the somatic fatigue component correlated with the inflammation in the group of non-depressed participants who by definition had no mood disorders^62^.

However, the dimensions of cognitive fatigue and inflammation correlated in both groups. This could mean that the correlation between pro-inflammatory cytokines (TNF-α in depressed participants, IL-6 in non-depressed participants) and cognitive fatigue symptoms may not be affected by depression. In many diseases without a primary mental component, cognitive fatigue symptoms also correlate with pro-inflammatory cytokines^16,29,63,64^.

Finally, the fatigue dimensions correlated in the HCG with various cytokines - TNF-α with cognitive fatigue as well as TNF-α and IL-6 with cognitive and somatic fatigue (see Table 5). Although they are different cytokines, both are produced by the macrophage and induce similar effects in the human body^57^.

**Table 5:** Spearman’s ρ correlation table.

		N	BDI-FS			COG-F			SOM-F			PSY-F		
			ρ	P	CI95	ρ	P	CI95	ρ	P	CI95	ρ	P	CI95
BDI-FS	PG	43	—			*0.48	0.0005	[0.26, 1]	*0.42	0.002	[0.19, 1]	*0.77	<0.0001	[0.64, 1]
	HCG	51	—			*0.46	0.0004	[0.25, 1]	*0.47	0.0002	[0.27, 1]	*0.45	0.0005	[0.45, 1]
IL-6	PG	42	0.23	0.08	[−0.03, 1]	0.27	0.04	[0.01, 1]	0.11	0.25	[−0.15, 1]	0.18	0.13	[−0.09, 1]
	HCG	51	0.12	0.20	[−0.12, 1]	*0.34	0.007	[0.12, 1]	*0.37	0.004	[0.15, 1]	0.31	0.01	[0.09, 1]
IL-1β	PG	42	0.24	0.07	[−0.02, 1]	0.11	0.24	[−0.15, 1]	−0.13	0.79	[−0.37, 1]	0.12	0.22	[−0.14, 1]
	HCG	51	0.06	0.34	[−0.06, 1]	0.25	0.04	[0.02, 1]	0.32	0.01	[0.09, 1]	0.28	0.02	[0.05, 1]
TNF-α	PG	43	*0.47	0.0008	[0.24, 1]	*0.36	0.0095	[0.11, 1]	0.32	0.02	[0.07, 1]	*0.40	0.004	[0.16, 1]
	HCG	51	−0.08	0.70	[−0.30, 1]	0.27	0.03	[0.04, 1]	*0.34	0.007	[0.12, 1]	0.24	0.05	[0.01, 1]
IFN-γ	PG	43	−0.22	0.93	[−0.45, 1]	−0.29	0.97	[−0.51, 1]	−0.37	0.99	[−0.57, 1]	−0.26	0.96	[−0.48, 1]
	HCG	51	0.13	0.19	[−0.11, 1]	−0.14	0.83	[−0.36, 1]	−0.31	0.99	[−0.51, 1]	−0.20	0.92	[−0.41, 1]
CRP	PG	39	0.01	0.49	[−0.26, 1]	−0.23	0.92	[−0.47, 1]	−0.15	0.82	[−0.40, 1]	−0.10	0.73	[−0.36, 1]
	HCG	46	0.18	0.12	[−0.07, 1]	−0.08	0.69	[−0.32, 1]	0.14	0.18	[−0.11, 1]	0.05	0.38	[−0.20, 1]

One-tailed-p-values under the Bonferroni corrected p-value (p < 0.01) were flagged (*).

Abbreviations: ρ = Spearman’s rho, CI95 = 95% confidence intervals, P = p-value, HCG = healthy controls group, PG = patient group, COG-F = Cognitive fatigue component scores (FIS-D); SOM-F = Somatic fatigue component scores (FIS-D); PSY-F = Psychosocial fatigue component scores (FIS-D); BDI – FS = Beck Depression Inventory – Fast Screening; IL-6 = Interleukin 6; TNF-α = Tumor necrosis factor alpha; IL-1β = Interleukin 1 Beta; IFN-γ = Interferon gamma; CRP = C-reactive Protein.

### Limitations

As always, the sample size could be larger to generalize the results beyond the context of the study. However, the power obtained from this study with 94 participants was 1-β = 0.91, value that overcome the 1-β = 0.80 threshold. The sample size for the study design used should therefore be sufficient to examine the expected effects.

The higher number of women compared to men in both groups examined could have influenced the results. In Germany, the ratio of women to men treated for depression is approximately 2: 1^65,66^. This relationship is also reflected in this study.

As expected, there were many very low BDI depression scores in the group of non-depressed participants, which led to a positively skewed distribution. Somehow surprisingly, no floor effects were seen for the fatigue values. However, IL-1β was positively skewed in both groups, TNF-α only in the group of depressed participants. This skewness was mainly due to the occurrence of some higher values. Rank correlation analyzes were therefore calculated where possible. However, MANCOVA’s results could be slightly affected.

CRP data being labeled as maximum values by the ‘absorbance plate reader’ in nine participants hat to be excluded from analysis (n_PG_ = 4, n_HGC_ = 5). CRP is an acute phase protein with anti-inflammatory effects, but is mainly non-specific, levels could be elevated and not necessarily be correlated with inflammatory processes^49^.

Smoking can affect cytokine levels. Therefore, smoking behavior was treated as a confounding factor, but it did not appear to affect the results of the current study.

### Future directions

There are several points of interest for future studies. Proinflammatory markers were sampled from the plasma but not obtained from CSF. It would be of advantage validate the results with central cytokine concentrations. Future studies could include cortisol and ACTH, both influenced by cytokines in the regulation of HPA activity, in the study of inflammation, depression, and fatigue. As always, longitudinal studies of cytokine levels in depression and fatigue-related illnesses are worthwhile to primarily determine whether cytokine levels are more likely in emergencies or in acute situations. In this research area, it is certainly an important goal to investigate whether the (first) onset of depression is also related to changes in the cytokine concentration and whether there is a connection between deregulation of HPA, inflammation and depressive symptoms. The extension of the methods to *in vivo* magnetic resonance spectroscopy would be of interest to establish a connection between the hypothalamic activity and the cytokine concentrations in the peripheral plasma or in the CSF.

## Conclusions

At the subjective self-assessment level, the study showed a clear difference in all dimensions of fatigue in depressed compared to non-depressed participants. The fatigue dimensions, but not the severity of the depression, showed differences in IL-6 concentrations. The pro-inflammatory cytokine IL-6 was also increased in participants with depression. Correlations within groups showed that the severity of depression determined by the BDI-FS was positively associated with TNF-α in depressed, but not in non-depressed participants. In addition, TNF-α correlated with psychological and cognitive fatigue within the group of depressed participants, but not in the control group. The correlation pattern was different in the group of non-depressed participants. IL-6 correlated with cognitive and somatic fatigue, TNF-α correlated only with somatic fatigue. In summary, correlations of fatigue and inflammation were found in both groups, but not equally for every dimension of fatigue in each of the groups. This could mean that the correlation between inflammatory cytokines and fatigue symptoms is not necessarily influenced by depression and therefore fatigue and depression are overlapping constructs, but are not identical. Future studies could include cortisol and ACTH, both of which are affected by cytokines in regulating HPA activity to address this issue.

## Methods

### Study design

A case-control study was conducted. 43 patients diagnosed with depression or depressive episode (DE) as defined by ICD-10 (PG) and 51 healthy controls (HCG; community sample), aged between 18 and 65 years, were included in the study. Groups were matched by age and gender. Data collection was performed between November 2018 and September 2019. In the PG, volunteers with DE but without concomitant psychotic episode were included. Patients having another comorbid psychiatric disease (i.e. bipolar disorder, personality disorder, adaptation syndrome and post-traumatic stress disorder) were included as long as a DE was present and predominant during the last 6 months. Exclusion criterion for both PG and HCG were insufficient German language knowledge, somatic or cognitive limitations that did not allow participation, particularly visual or auditory limitations. Individuals could not participate if they suffered from acute or chronic disease or illness of any type, particularly related to infection, except depression. Latter were assigned to the patient’s group. Demographic data are shown in Table 1.

Participants or legal authorized representatives were informed about the procedure and gave written informed consent of participation. All experimental procedures were in accordance with the Declaration of Helsinki and were approved by the local ethics committee of the Justus-Liebig University (JLU) medical faculty. The study complies with the APA ethical standards.

### Data collection

#### Blood Sampling

3 mL fasting venous blood samples were collected between 8:00 am and 12:00 pm with EDTA K blood sample tubes (S-Monovette 2.7 mL K3E tube with 1.6 mg EDTA/mL, SARSTEDT AG & Co. KG, Nümbrecht, Germany) and then centrifuged at 4 °C with 1100 × g for 15 minutes. After centrifugation, the plasma was collected, divided into two 0.5 mL aliquots and immediately stored at −20 °C. Every 4 weeks, the blood samples were collectively delivered to an university research facility, which was about 30 km away, and stored at −80 °C for further use.

Levels of IL-6, IL-1β, C-reactive Protein (CRP), Tumor necrosis factor-alpha (TNF-α) and Interferon-gamma (IFN-γ) were measured using ELISA (Quantikine ELISA kits, R&D Systems Inc., Minneapolis, Minnesota, United States of America) with the detection limits of: IL-6 = 3.13 pg/mL, IL-1β = 3.9 pg/mL, CRP = 0.78 ng/mL, TNF-α = 15.6 pg/mL and IFN-γ = 15.6 pg/mL. Intra- and inter-precision values were <10%.

IL-1β values below the ELISA detection limit (one participant) were considered to be zero pg/mL and included in the analysis. Maximum plasma values (indicated by the software as ‘>Max’) for IL-6 (one participant), CRP (nine participants) and IL-1β (one participants) were reported and excluded from the analysis.

Pro-inflammatory markers’ concentrations were calculated using the Tecan Reader and Magellan Reader Software (Tecan Group Ltd., Männedorf, Switzerland). For the parameter calculation, the Marquardt’s 4-parameter estimation method was used.

#### Depression

DE as defined according to the diagnostic criteria from the International Statistical Classification of Diseases and Related Health Problems, 10^th^ version (ICD-10)^3^. The presence of a DE was diagnosed in the psychiatric department of the University Hospital of Giessen and Marburg (Location - Giessen), by clinical experts.

The Beck Depression Inventory – Fast Screening, German Version (BDI-FS)^67^ was applied for scoring the severity of the DE. The BDI-FS provides scores in the range between 0–21. The highest score indicates a high load of depressive burden. The instrument has good internal consistence (Cronbach’s α = 0.84) and a convergent validity with the PHQ-9 of r = 0.67, including that it was validated to a representative German sample (n = 2467). If necessary, the cut-off definitions recommended in the manual were used (cut-off = 5). The categories used were labeled as *minimal* (0 to 3 points), *mild* (4 to 8 points), *moderate* (9 to 12 points), and *severe* (13 to 21 points).

#### Fatigue dimensions

The extent of fatigue was assessed on the subjective level. The subjective ratings of fatigue were recorded applying the Fatigue Impact Scale – German Version (FIS-D^68^) in both groups. The FIS-D measures the impact of fatigue on Health-Related Quality of Life^68^. The FIS-D consists of three sub-scales, which represent a three-dimensional structure of fatigue. These dimensions include a psychosocial dimension (or PSY-F, 20 questions, maximum score 80 points), a somatic dimension (or SOM-F, 10 questions, maximum score 40 points) and a cognitive dimension (or COG-F, 10 questions, maximum score 40 points). The maximum score that can be acquired is 160 points (4 for each item). As defined in the FIS-D manual, the cut-off values for increased fatigue are over 20 points for the psychosocial dimension, over 10 points for the somatic or cognitive dimension, and over 40 points for the full test.

### Statistical Analysis

Statistical analyses were performed using SPSS version 24.0 (Statistical Package for the Social Sciences, International Business Machines Corporation, New York, United States of America) and JASP version 0.11.1 (Jeffreys’s Amazing Statistic Program, The University of Amsterdam, Amsterdam, The Netherlands).

Quantitative variables approximately fitting a normal distribution are specified in the text as mean ± standard deviation (M ± SD), those with a non-normal distribution were expressed as median (Me) with percentile 75 (Q3) and percentile 25 (Q1) and the interquartile range (Q3–Q1; IQR). Categorical variables were specified with numbers and in some cases quotient values. For obtaining results with two decimals, data was rounded to the next decimal. Values smaller than 0.001 were denotated as <0.001 and values greater than one million were expressed in scientific notation.

To improve readability, the data was organized in tables. General sample information is listed in Table 1 and information regarding psychological and immunological parameters is displayed in Table 2.

Table 1 also shows the group difference statistics. The Student t-test was used for continuous, parametrically distributed data. Otherwise, the U-Mann-Whitney test was used. For group differences in categorical data, χ2 or the exact Fisher test was calculated. Then homogeneity between groups (i.e. PG = HCG) was assumed if the bilateral p-value was greater than a threshold value of 0.05.

Regarding the information for the effect sizes on the Table 1, Cohen’s d (for parametrical distributed data), Eta-Square (for non-parametrical distributed data), and Cramer’s V (for categorical data). Cohen’s d thresholds were defined as low = 0.2, medium = 0.5, high = 0.8. For the Cramer’s V effect size, we used the following thresholds: very low 0 to 0.1, low 0.1 to 0.3, medium 0.3 to 0.5 and high up to 0.5. For the non-parametrical data, Eta-Square (ES) was used under the following formula: η^2^ = Z^2^/N-1. The effects were defined as weak whenever ES ≤ 0.04; ES was defined as medium when 0.04 < ES ≤ 0.36 and as strong when ES > 0.36.

For the mean comparisons between the groups, two multivariate analyses of covariance (MANCOVA) were computed, one for the psychological variables (PSY-F, COG-F, SOM-F) and the other for the pro-inflammatory cytokines IL-6, IL-1β, TNF-α, IFN-γ, and CRP. *Gender*, *age*, and *BMI* were included in both models as covariates. These results are shown in Tables 3 and 4. The mean differences between groups were flagged ‘significant’ if the two-tailed-p-value was smaller as 0.05. Partial eta-square values were calculated for the effect sizes. For the multivariate tests, the partial ES was calculated using the following formula: η^2^_p_ = (df_1_ * F)/[(df_1_ * F) + df_2_]^69,70^. For the univariate test, the partial ES was calculated using the following formula: η^2^_p_ = (SS_effect_)/[SS_effect_ + SS_error_]^69,70^. The effects for both partial ES formulae were defined as following: very small (η^2^_p_ < 0.01), small (0.01 ≤ η^2^_p_ < 0.06), moderate (0.06 ≤ η^2^_p_ < 0.14) and large (η^2^_p_ ≥ 0.14)^69,70^.

For the correlation matrices within each group, Spearman rank correlations (Spearman’s ρ) between all variables including the total BDI-FS score were calculated. All variables were previously adjusted for the influence of the confounding factors *gender*, *age*, *smoking-behavior*, *medication intake*, and *BMI*. For this purpose, a linear model was calculated for each variable with the confounding factors as predictors for both groups separately. The residuals were used as new variables. According to the hypothesis that the higher the pro-inflammatory cytokine concentrations, the higher the fatigue dimension or the depression values, only positive correlations were considered and therefore one-sided tests were calculated. In order to take multiple testing into account, corresponding p-values were compared with the threshold value at α = 0.05, which Bonferroni was adjusted by the number of pro-inflammatory markers. In this way, a threshold value of p = 0.01 was obtained, which was defined as significant for this method.

### Ethical Approval and Consent to participate

This study was approved by the ethic committee of the JLU medical faculty (Annex 1). Additionally, this study is part of a big project to investigate inflammatory factors and fatigue in patients with depression and multiple sclerosis. The code of this project in the ethic committee is AZ 81/18. Attached is the ethical approval and the informed consents (Annex 2, Annex 3) in its original language (German).

## Data Availability

The data that support the findings of this study are not publicly available due to the approved law of data protection from the European Union but are available from the corresponding author on strictly grounded reasonable requests.
